# Supplementary material for: Rac1 GTPase activates the WAVE regulatory complex through two distinct binding sites
Source: eLife. 2017 Sep 26;6:e29795. doi: 10.7554/eLife.29795 (PMC5614565; doi:10.7554/eLife.29795)
Supplement: Supplementary file 2. [file elife-29795-supp2.docx]

Supplementary File 2

**Scripts used in the DynaFit program to fit the equilibrium pull-down data.** Note that the scripts will not be recognized by DynaFit if directly copied from this Word file and pasted into the program. The scripts need to be first copied into a plain text editor to remove hidden characters from the Word file.

;----------- single site model-------------

[task]

task = fit

data = equilibria

confidence = monte-carlo

algorithm = LM

[settings]

{MonteCarlo}

Runs = 5000

RandomizationMethod = shuffle

[mechanism]

;L for Rac, P for WRC

L + P <===> LP : Kd1 dissoc.

[constants]

Kd1 = 0.2?

[concentrations]

P = 0.1

[responses]

LP = 10

[equil]

directory ./_New_Fig5

extension txt

variable L

file WT_data

plot logarithmic

[output]

directory ./_New_Fig5/WT_model1_out

[end]

;----------- two site cyclic model-------------

[task]

task = fit

data = equilibria

confidence = monte-carlo

algorithm = LM

[settings]

{MonteCarlo}

Runs = 5000

RandomizationMethod = shuffle

[mechanism]

;L for Rac, P for WRC, (LP)D for Rac bound to D site of WRC, (LP)A for Rac bound to A Site

L + P <===> (LP)D : Kd1 dissoc.

L + P <===> (LP)A : Kd2 dissoc.

L + (LP)D <===> (LP)AD : Kd3 dissoc.

[constants]

Kd1 = 0.2?

Kd2 = 1?

Kd3 = 0.1?

[concentrations]

P = 0.1

[responses]

(LP)D = 10

(LP)A = 10

(LP)AD = 10

[equil]

directory ./_New_Fig5

extension txt

variable L

file WT_data

plot logarithmic

[output]

directory ./_New_Fig5/WT_model4_out

[end]

;----------- two site sequential model-------------

[task]

task = fit

data = equilibria

confidence = monte-carlo

algorithm = LM

[settings]

{MonteCarlo}

Runs = 5000

RandomizationMethod = shuffle

[mechanism]

;L for Rac, P for WRC

L + P <===> LP : Kd1 dissoc.

L + LP <===> L2P : Kd2 dissoc.

[constants]

Kd1 = 0.2?

Kd2 = 1?

[concentrations]

P = 0.1

[responses]

LP = 10

L2P = 10

[equil]

directory ./_New_Fig5

extension txt

variable L

file WT_data

plot logarithmic

[output]

directory ./_New_Fig5/WT_model2_out

[end]

;----------- GST dimerization model-------------

[task]

task = fit

data = equilibria

confidence = monte-carlo

algorithm = LM

[settings]

{MonteCarlo}

Runs = 5000

RandomizationMethod = shuffle

[mechanism]

;L for GST-Rac (concentration converted to half of Rac concentration), P for WRC. Derived Kd1 need to multiply by 2 to obtain Kd1 for Rac1/WRC.

L + P <===> LP : Kd1 dissoc.

LP + P <===> LP2 : Kd2 dissoc.

[constants]

Kd1 = 0.2?

Kd2 = 1?

[concentrations]

P = 0.1

[responses]

LP = 10

LP2 = 10

[equil]

directory ./_New_Fig5

extension txt

variable L

file WT_data_GSTdimer

plot logarithmic

[output]

directory ./_New_Fig5/WT_model3_out

[end]
